# THE SOCIAL RELATIONSHIP OF OLD ADULTS ACCORDING TO THE USE OF ASSISTIVE APPLIANCES IN SOUTH KOREA.

**DOI:** 10.1093/geroni/igad104.3664

**Published:** 2023-12-21

**Authors:** Yeojin Yoon

**Affiliations:** Chung-Ang University, Seoul, Republic of Korea

## Abstract

This study aims to examine the differences in the social relationship of the old adults between the use of assistive appliances. For the analysis, the data were obtained from “2020 National Data on the Elderly” published by the Ministry of Health and Welfare and the Korea Institute for Health and Social Affair. Data was a survey of people aged 65 and older conducted between June and August 2020, which included data from 10,097 people. The variables used for the use of assistive appliances were vision aids, hearing aids, chewing aids. The variables used to measure older adults’ social relationship resource are the frequency of visits and contact with non-resident children, grandchildren and children with whom they have the most contact, and the frequency of visits and contact with relatives, friends, neighbors, and acquaintances. The analysis used multiple regression analysis and t-test for comparison of means between groups, and the main findings were as follows. First, there is no statistical correlation between assistive appliances use and older adults’ social relationships. Second, there is no significant difference in the mean of the social relationships of older adults with and without assistive appliances use. In this paper, we overlooked the ease with which the elderly can use assistive appliances with support if they have functional difficulties. These findings confirm that there is no direct relationship between the use of assistive appliances and the social relationships of older adults, and suggest the need for further research on the social relationships of older adults.

